# Obesity and Headache/Migraine: The Importance of Weight Reduction through Lifestyle Modifications

**DOI:** 10.1155/2014/420858

**Published:** 2014-04-03

**Authors:** Alberto Verrotti, Alessia Di Fonzo, Laura Penta, Sergio Agostinelli, Pasquale Parisi

**Affiliations:** ^1^Department of Pediatrics, University of Perugia, Località S. Andrea delle Fratte, 06132 Perugia, Italy; ^2^Department of Pediatrics, University of Chieti, Italy; ^3^Pediatric Unit, Ospedale Madonna del Soccorso, San Benedetto del Tronto (AP), Italy; ^4^Department of Child Neurology, II Faculty of Medicine, “La Sapienza” University, Rome, Italy

## Abstract

The aim of this study is to determine a possible relationship between prevalence, frequency, and severity of migraine and obesity. All pertinent data from the literature have been critically examined and reviewed in order to assess the possible relationship between obesity and migraine, in particular migraine frequency and disability in children, as well as in adult population studies. Prevalence, frequency, and severity of migraine appear to increase in relation to the body mass index, although this evidence is not supported by all the studies examined. Data from literature suggest that obesity can be linked with migraine prevalence, frequency, and disability both in pediatric and adult subjects. These data have important clinical implications and suggest that clinicians should have a special interest for weight reduction of obese children suffering from migraine, prescribing and supporting intensive lifestyle modifications (dietary, physical activities, and behavioral) for the patient and the entire family.

## 1. Introduction

Headaches are common during childhood and become more common and more frequent during adolescence. International headache society (IHS) divides headache into primary and secondary headache disorders. Primary headaches comprise migraine, tension-type headache, cluster headache, other autonomic cephalgias, and other primary headache disorders.

Migraine is defined by the IHS as a recurrent headache that occurs with or without aura and lasts 4–72 h. Migraine is best understood as a chronic disorder with episodic manifestations, progressive in some individuals. Consistently, identifying risk factors for progression has emerged as a very important public health priority.

Recent studies suggested a potential role of obesity on migraine outcomes. Obesity occurs with several chronic pain syndromes. Obesity and migraine are both highly prevalent disorders in the general population, and reports in the literature underscore this association. An increasing number of reports suggest that obesity is a risk factor for migraine progression and migraine frequency in adults [1–4] as in children [5–7].

The aim of this review was to summarize and critically appraise evidence from the most recent clinical and population-based studies, evaluating the possible association between obesity and migraine; we also evaluated if the reduction of BMI through modifications of the lifestyle should be a therapeutic approach to reduce frequency and severity of headache/migraine.

Headache and obesity are prevalent and disabling disorders that are influenced by a variety of physiological, psychological, and behavioral mechanisms, many of which are affected by weight loss [8]. It is not unusual for migraineurs patients to be obese. Recently, attention has focused on the potential relation between overweight and frequency and severity of headache attacks [9, 10] and some evidence for this relationship has been demonstrated [11, 12].

One of the largest population studies emphasizes the association between obesity and chronic daily headache (CDH) although it underlines that this association was relatively specific only for chronic migraine (CM) [13]. The question of the link between obesity and migraine frequency is still matter of debate while the majority of studies have suggested a certain influence of the overweight of the subjects on migraine severity.

## 2. Epidemiological Relationship between Headache/Migraine and Obesity

A PUBMED search of the English-language studies published between 2000 and 2012 investigating the possible association between migraine and obesity was performed; key terms used alone or in combination included: migraine, obesity, overweight, and body mass index.

Specific review articles and systematic reviews were examined for any further publications, as were the reference sections of all articles identified by literature search. Validation was undertaken by a second review of the search results to ensure that no article had been missed.

The main studies that addressed this relationship are reported in Table 1.

One of the first studies to identify an association between frequent headache and obesity was a study involving 1932 patients, aged 18–65 years, by Scher and colleagues in 2003 [15]. The population studied included 1134 patients who were CDH sufferers and 798 who had episodic headache. The most important result was that the prevalence of CDH was associated with total body obesity (OR 1.34; CI: 1.0–1.8) or overweight (OR 1.26; 1.0–1.7). Moreover, individuals with episodic headache who also had total body obesity at baseline were at increased odds of having CDH at follow-up (OR 5.28; CI: 1.3–21.1).

More in detail, an important percentage (30%) of newly identified cases of CDH showed clearly obesity, while only 13% of patients with episodic headache were obese. The result of this study is that individuals with episodic headache and obesity develop CDH more than 5 times the rate of normal-weight individuals.

Similarly, Ohayon [16] and colleagues found that overweight/obese (BMI > 27) respondents were more likely to report morning headache than were adults with BMI 20–25 and among a sample of ~15,000 Australian women, Brown and colleagues [14] found that obese persons were more likely to report headache (OR = 1.47), confirming again the association between headache and obesity.

In 2005, two small clinic-based studies reported an increased frequency of migraine attacks in those with total body obesity (TBO). In the first study the relationship between migraine and obesity was evaluated and it showed that obese patients were three times as likely as age-matched normal-weight controls to have migraine; in fact Prieto Peres et al. [4] compared 74 patient with TBO (mean age 39 years) who presented obesity surgery clinic to 70 age-matched controls. A total of 75% of those with TBO had life-time headache diagnosis as compared with 42% of the controls, *P* < 0.001. Furthermore, CDH migraine was compared with 18.5% of the nonobese controls, *P* < 0.0001. Similarly, in the second clinic-based study by Horev et al. [23], 63% of 27 patients with TBO reported episodic headache and 48% fulfilled migraine criteria. The results of those studies showed that migraine was the most common diagnosis and was as prevalent in obesity grade III as in overweight and obesity grades I and II.

Bigal et al. [9] showed that obesity was not associated with increased prevalence of migraine but was related to headache attack frequency. In this population-based telephone interview study, the subjects were subdivided into five groups, considering their BMI: 1, underweight (<18.5), 2, normal weight (18.5 to 24.9), 3, overweight (25 to 29.9), 4, obese (30 to 34.9), and 5, severe obese (≥35): the odds ratio for headache frequency increased significantly from group 1 to group 5. There was a robust evidence that groups 3, 4, and 5 migraineurs showed a high risk for having great and frequent headache; in contrast, groups 1 and 2 subjects did not show this risk.

On the other side, another large cross-sectional population research added more evidence about the association between obesity and CDH and demonstrated that obesity is an important determinant for CM but not for chronic tension type headache [13].

The same authors showed that CDH and total body obesity were more significantly associated in transformed migraine than in chronic tension-type headache (CTTH).

The objective of Ford's work [11] was to study the cross-sectional association between body mass index and the prevalence of severe headaches or migraines in a national sample of US adults. They evaluated 7601 participants in the national Health and Nutrition Examination survey (NHEANES), ranging from 20 to 85 years of age. Migraine and severe headache were self-reported, showing that those who were underweight (BMI < 18.5) or obese (BMI > 30) were at higher risk for having severe headaches or migraine compared with those of normal weight.

In the same year Pinhas-Hamiel et al. [7] in a prospective cohort study confirmed these results of Ford and coauthors; in fact this study [7] demonstrated positive correlations between frequency of migraine and obesity.

A total of 21.783 participants were included in the Peterlin's analysis [12] in order to evaluate the prevalence of migraine/severe headaches in those with and without general obesity and abdominal obesity (Abd-O) and the effect of gender and age on this relationship. They found in men and women aged 20–55 years that higher migraine prevalence was associated with both total and abdominal obesity. And, this was the first study which suggested and clearly demonstrated that older individuals or those of postreproductive age who have migraine do not have an association with obesity while those of reproductive age do, which also suggested that both obesity and migraine are modulated by reproductive status. The finding suggests that migraine and obesity are associated in those subjects in reproductive age [12]; this association was also later supported by data from Vo et al. [20] and Robberstad et al. [24]. Vo et al. found a significant association between migraine and obesity and that the odds of migraine increased with increasing obesity status. Robberstad et al. found that recurrent headache was associated with overweight (odds ratio [OR] = 1.4, 95% CI 1.2–1.6, *P* = 0.0001).

The relationship between obesity and headache/migraine has not been adequately studied with pediatric populations. The first study to examine the prevalence of obesity within a pediatric headache population was the Hershey's work [5] in which were examined 913 patients at 7 pediatric headache centers and the results clearly showed that obesity was significantly correlated with headache frequency and disability in children, and reduction in BMI as associated with greater reduction in headache frequency. Interestingly, the degree of overweight (measured by BMI percentile) correlated with both headache frequency and disability of headache. Of a certain relevance is the fact that the magnitude of weight reduction was related with decreased headachefrequency at 3- and 6-month follow-up visits.

In another pediatric study, Kinik et al. [6] investigated the influence of obesity on the severity of migraine in children. In agreement with previous adult study the authors concluded that obesity seems to occur at greater frequency in children and adolescents with migraine compared with the general population, and obese patients had more frequent migraine attacks than did nonobese patients.

Recently in a retrospective study [25] of 925 children in the Pediatric Headache Clinic, evaluating headache frequency, medication overuse, and BMI compared to population-based healthy subjects, children with headache had a greater percentage of overweight in comparison with the general population. It should be noted that also the patients with chronic tension-type headache showed similar results. On the other hand, there was no increased incidence of overweight in children with medication overuse or chronic migraine. It is important to remember that in adult series [13], a link between chronic migraine and obesity was found but with chronic tension-type headache.

Nevertheless, not all studies found the positive correlation between migraine and obesity. In fact Mattsson [19] failed to detect a significant correlation between obesity and migraine in 684 women aged 40–74 years. Similarly, in the study of Keith et al. [17], migraine prevalence was not related to obesity but obese women (BMI of 30) had increased risk for headache (but not specifically migraine) as compared with those with BMI. Téllez-Zenteno et al. [26] found that there was no association between the disability and severity of migraine and BMI, as well as no correlation between BMI and the frequency and prevalence of migraine was found in the study of Bigal et al. [18] in which 176 subjects (79.5% women, mean of 44.4 years) with normal weight (≤ 24.9), overweight (25–29.9), or obesity (≥30) were observed before and after headache preventive treatment. After treatment, frequency declined in the entire population, but no significant differences were found by BMI group.

Regarding the number of days with severe pain per month, there were also no significant differences at baseline (normal = 6.1, overweight = 6.5, obese = 6.7), and improvement overall (*P* = 0.01). Recently, also Winter et al. [22] in their large prospective study of middle-aged women do not indicate a consistent association between migraine and incident overweight, obesity, or relevant weight gain.

In Yu et al.'s [21] work they had shown that there is an association between morbid obesity and migraine in Asian population, while in the studies of Peterlin et al. [12] and Vo et al. [20] this relation was found in a Caucasian population. In this study it was found that migraine prevalence was significantly raised in the morbidly obese group (but not lesser degrees of obesity), and this was a substantial and statistically significant increase, but they also observed that there was a weak link between being underweight and migraine severity and disability.

## 3. Obesity-Related Associated Comorbidities: Lifestyle Intervention to Reduce BMI

Childhood obesity and overweight can be related to some adult diseases, in order to be predictive in adult life of obesity and overweight; it is well known that cardiovascular risk factors increase with the rise in BMI. The presence of cardiovascular risk factors during childhood can lead to an enlarged incidence of fatal and nonfatal cardiac events in adulthood.

In pediatric age, diseases associated with obesity are also possible. The pediatric obesity-associated comorbidities already described are type 2 diabetes mellitus (T2DM); dyslipidemia (most commonly a low HDL cholesterol); metabolic syndrome; hyperandrogenemia and hyperinsulinism in pre- to midpubertal girls and consequent polycystic ovary disease (PCOS); systolic blood pressure; proteinuria and focal segmental glomerulosclerosis; obstructive sleep apnea; nonalcoholic fatty liver disease (NAFLD); gallstones; orthopedic pathologies; pseudotumor cerebri; and finally, psychosocial problems [27].

The key determinants of childhood obesity in developing countries are unhealthy nutrition with increased caloric intake, reduced physical activity, urbanization, residence in metropolitan cities, socioeconomic status and sociocultural factors, age, and female gender. Therapeutic lifestyle changes and maintenance of regular physical activity through parental initiative and social support interventions are the most important strategies to challenge childhood obesity [28]. Lifestyle changes should include healthy eating habits (avoiding the consumption of calorie-dense and nutrient-poor foods, eating adequate portion, increasing intake of dietary fiber, fruits, and vegetables, also in school meals, eating timely, particularly breakfast and avoiding constant “gazing” during the day); physical activity (performing 60 minutes of daily moderate to vigorous physical activity, also in schools, walking and cycling to school, decreasing time spent in sedentary activities, such as computer/TV time); parents education (healthy culture patterns related to diet and activity, explaining the caloric needs and essential nutrient requirements of young children) [27].

Childhood obesity is a grave issue that needs to be addressed urgently: the objective of interventions in overweight and obese children and adolescents is the prevention or amelioration of obesity-related comorbidities, for example, glucose intolerance and T2DM, metabolic syndrome, dyslipidemia, and hypertension [28] and finally also headaches/migraine, because we can consider headache/migraine as another obesity-associated comorbidity.

## 4. Conclusions

Migraine is a chronic neurological disorder characterized by recurrent attacks of pain that generally impair the quality of life. The real etiology and pathogenetic mechanism(s) of migraine are still unknown. Obesity is another chronic disorder that is very frequent both in adult and in pediatric population. Although a clear comorbidity between these conditions has been recently demonstrated, the real link between them is still matter of debate. Although this comorbidity is now recognized, the basic nature of this association is still unclear; it is possible that migraine and obesity can have some common pathophysiologic mechanisms and share one or more final pathways (e.g., inflammatory mediators). Obesity (and often body mass index of the patients) seems to be related not only to high frequency and to the degree of migraine attacks (especially some types of migraine) but also to the prevalence of the latter. These relations seem to be present in both adult and paediatric subjects. These relations are important for clinical practice and for future research. Given the association between obesity and headache, clinicians should actively consider a child's weight status in the context of treatment for headache. Routine assessment of child weight using BMI percentiles should be undertaken at the initial visit and used in the conceptualization of the presenting problem. For children who are overweight or at risk for overweight at the beginning of treatment, educational intervention may be necessary to improve weight control and subsequent headache treatment outcomes. For some children, referrals for behavioral weight management services may be necessary to facilitate appropriate lifestyle changes (increasing exercise, improving adherence to dietary guidelines) for effective weight control and optimal headache management.

## Figures and Tables

**Table 1:** Characteristics and main results of studies concerning obesity and migraine.

Reference	Clinical study design	*N*.	*F*. (%)	Age (average age or range)	Characteristics of migraine or headache prevalence frequency and severity	
Brown et al. [14]	Cohort study	14779	100	18–23	Women in the highest BMI category were more likely to report headaches	Deleterious effects of overweight also for headaches
Scher et al. [15] Pain 2003	Longitudinal study	1932	NA	18–65	Prevalence of CDH was higher in those with TBO > grade II	Obesity and headache frequency were significantly associated with new onset CDH
Ohayon [16]	Observational study	18980	51.3	15 years or older	The prevalence of morning headaches was linked to BMI	A higher prevalence of morning headaches in subjects with BMI less than 20 or >27 than in subjects with normal BMI
Prieto Peres et al. [4] Arq Neuropsiquiatr 2005	Case-control study	74	89	38.4 (14–69)	Increased attacks of severe headache in obese compared with normal weight. Increased prevalence of migraine in obese women than obese men	No correlation between frequency of migraine and obesity. Severity: NA
Bigal et al. [9] Neurology 2006	Observational cohort study	30.125	65	38.7	No correlation between the prevalence of migraine and obesity	Positive correlation between migraine frequency and obesity Severity: NA
Keith et al. [17] Obesity 2006	Cross-sectional analysis	220.370	100	16–94	No correlation between the prevalence of migraine and obesity	NA
Bigal et al. [18] Cephalalgia. 2006	Longitudinal study	176	79.5	44.4	No correlation between the prevalence of migraine and obesity	No correlation between frequency/severity of migraine and obesity
Bigal et al. [10] Arch Intern Med. 2007	Observational cohort study	162.576	NA	≥12	NA	Positive correlation between migraine frequency and obesity Severity: NA
Mattsson et al. [19] Cephalalgia 2007	Cross-sectional analysis	684	100	40–74	No correlation between the prevalence of migraine and obesity	No correlation between frequency/severity of migraine and obesity.
Pinhas-Hamiel et al. [7] Obesity 2008	Prospective cohort study	273	61	13 (9–17)	Increased headaches in overweight girls compared with normal weight	Positive correlation between frequency of migraine and obesity. Severity: NA
Ford et al. [11] Cephalalgia. 2008	Cross-sectional analysis	7.601	48	≥20	Positive correlation between the prevalence of headache and obesity	Increased attacks of severe headache in overweight or obese when compared with normal weight.
Hershey et al. [5] Headache 2009	Large, multicenter, retrospective case	913	59	11.9 (3–18)	Increased attacks of severe headache in overweight or obese compared with normal weight	Positive correlation between frequency/severity of migraine and obesity Severity: NA
Peterlin et al. [12] Headache 2010	Cross-sectional analysis	21.783	51	≥20	Increased prevalence of migraine in subjects aged <55 with total or abdominal obesity	NA
Kinik et al. [6] Cephalalgia 2010	Cross-sectional analysis	124	62	12.9 (4–17)	Increased attacks of severe headache in overweight or obese compared with normal weight	Positive correlation between frequency/severity of migraine and obesity. No correlation between severity of migraine and obesity.
Vo et al. [20] Headache. 2011	Cross-sectional study	3733	100	18–40	Increased prevalence of migraine in patients with morbid obesity	Positive correlation between migraine severity and obesity
Yu et al. [21] J Headache Pain 2012	Cross-sectional analysis	5041	50	43.6	Increased prevalence of migraine in patients with morbid obesity	No correlation between frequency/severity of migraine and obesity
Winter et al. [22] Cephalalgia 2012	Prospective cohort study	19162	100	50	No correlation between the prevalence of migraine and obesity	No correlation between frequency/severity of migraine and obesity
